# Average physical structure of cratonic lithosphere, from thermodynamic inversion of global surface-wave data

**DOI:** 10.1007/s00710-025-00926-0

**Published:** 2025-06-17

**Authors:** Yihe Xu, Sergei Lebedev, Javier Fullea

**Affiliations:** 1 Department of Geophysics, School of Earth Sciences, Yunnan University, Green Lake North Road 2, Kunming, 650091 Yunnan China; 2 Department of Earth Sciences, University of Cambridge, Madingley Road, Cambridge, Cambridgeshire CB3 0EZ UK; 3 Department of Physics of the Earth and Astrophysics, Universidad Complutense de Madrid (UCM), Avda. de Séneca 2, 28040 Madrid, Spain

**Keywords:** Cratonic lithosphere, Geotherm, Seismic thermography, Lithosphere-asthenosphere boundary, Surface wave

## Abstract

Seismic and mantle-xenolith data both show that cratons have the thickest, coldest lithosphere of the Earth’s tectonic environments. Yet, the quantitative depth distributions of temperature, seismic velocity and mass density (herein referred to as “density”) in cratonic lithosphere are uncertain, even on average across all cratons. Seismic surface-wave data offer abundant information on the thermal structure of the lithosphere at present, but seismic-velocity profiles in tomographic and other seismic models are highly non-unique at the relevant depth scale lengths of tens of kilometres. Here, we relate surface-wave measurements averaged over all cratons globally to the average physical properties of cratonic lithosphere using the recently developed methods of seismic thermography. The thermodynamic inversion of the Rayleigh and Love wave phase-velocity curves yields a model of the average structure of cratonic lithosphere, including the profiles of temperature, S- and P-wave seismic velocities, density and radial seismic anisotropy. Average depleted peridotite composition of the cratonic lithosphere was taken from the literature. Assuming 1290 °C as the temperature at which convection commences, which defines the bottom of the mechanical lithosphere, the best-fitting average depth of the cratonic lithosphere-asthenosphere boundary (LAB) is 228 km, with an uncertainty range of 211–242 km, as estimated using the model-space projection approach. The model fits the observed phase velocities very closely (misfits < 0.055%), while also matching the observed topography and surface heat flow. Assuming a lower LAB temperature results in a shallower LAB and a broader transition from the conductive lithospheric geotherm to the mantle adiabat, with a similar fit to the data. Our craton-average lithospheric model offers a useful reference for geophysical studies and for the joint analysis of geochemical and geophysical data. It confirms that cratonic lithosphere is, on average, isopycnic: cratonic and non-cratonic upper-mantle density profiles are very similar. A large proportion of published pressure–temperature measurements from mantle xenoliths is close to our craton-average lithospheric geotherm. Many of the measurements from below 150 km depth, however, show temperatures significantly higher than cratonic average, which offers useful evidence on the evolution of cratons and generation of kimberlites.

**Supplementary Information:**

The online version contains supplementary material available at 10.1007/s00710-025-00926-0.

## Introduction

Cratons are characterised by cold, thick lithosphere, mostly Archean crust, and stability over billions of years (e.g., Pearson et al. 2021). The relatively cold geotherm beneath cratons translates into the greater thickness and mechanical strength of their lithosphere (e.g., McKenzie et al. 2005; Fullea et al. 2021; Afonso et al. 2022). The structure and temporal evolution of cratonic lithosphere control the distribution of diamondiferous kimberlites (e.g., Boyd et al. 1985; Giuliani et al. 2023), carbonatites (e.g., Gibson et al. 2024), sediment-hosted base metal deposits (e.g., Hoggard et al. 2020; Dou et al. 2024) and magmatic deposits (e.g., Griffin et al. 2013) as well as tectonic processes and the plate-tectonic evolution of continents.

A representative average physical model of cratonic lithosphere, including the profiles of temperature, density and seismic velocities is desirable in many problems in different fields of Earth science, including as reference in seismic and other geophysical imaging studies, geodynamic modelling, and in the studies of lithospheric composition and kimberlite dynamics (e.g., James et al. 2004; Cammarano and Romanowicz 2007; Schutt and Lesher 2010; Russel et al. 2012; Anzulovic and Caracas 2024; Lebedev et al. 2024b). For example, using a representative, craton-specific reference model in seismic imaging of cratons helps one to avoid common biases that occur when using a global-average seismic reference model (e.g., Dziewonski and Anderson 1981; Kennett et al. 1995) as reference (Davison et al. 2023).

Seismic data provide abundant information on the thermal structure of the lithosphere, thanks to the strong sensitivity of seismic wave speeds to temperature (e.g., Sobolev et al. 1996; Goes et al. 2000; Cammarano et al. 2003). Surface waves, in particular, are our richest source of information on the thermal structure of the mantle at lithospheric depths (e.g., Fullea et al. 2021; Civiero et al. 2024; Lebedev et al. 2024a). The rapid recent growth in the amount of broadband seismic data recorded around the world has resulted in a dense sampling of the globe with surface waves (e.g., Moulik et al. 2022). Yet, tomographic and other seismic models produced by the inversion of these data suffer from substantial non-uniqueness and cannot offer direct quantitative inferences on the lithospheric temperature and thickness, with their radial gradients at the relevant tens-of-kilometres scales within the lithosphere depth range highly uncertain (e.g., Civiero et al. 2024; Davison et al. 2023).

Seismic data can also be linked to temperature at depth using petrological relationships or physical constraints on seismic inversions (Furlong et al. 1995; Goes et al. 2000; Cammarano et al. 2003; Shapiro and Ritzwoller 2004; Priestley and McKenzie 2006; Afonso et al. 2008; Fullea et al. 2009; Khan et al. 2013; Deschamps et al. 2019; Shinevar et al. 2023; Priestley et al. 2024). Recently developed methods for the thermodynamic inversion of seismic data (e.g., Fullea et al. 2021; Xu et al. 2023) use computational petrology (e.g., Connolly 2005) and thermodynamic databases (e.g., Stixrude and Lithgow-Bertelloni 2011) to relate seismic and other data directly to mantle temperature and composition. Seismic-velocity sensitivity to composition is much weaker than that to temperature: compositional variations in the upper mantle change seismic velocities by, normally, less than 1%, at most 2% (e.g., Sobolev et al. 1996; Goes et al. 2000; Cammarano et al. 2003; Schutt and Lesher 2006), whereas the shear-wave speed variations in tomographic models are around ± 10% at the scales of a few hundred km, with most of them due to temperature variations (e.g., Schaeffer and Lebedev 2015). We can thus invert seismic data primarily for temperature, with assumptions on composition based on the consensus geochemical models. Thus formulated seismic thermography yields more accurate thermal models and, also, more accurate seismic models than purely seismic inversions, thanks to its ability to integrate relevant prior information and focus on the parts of the model space corresponding to physically plausible geotherms (Lebedev et al. 2024a).

Here, we use the thermodynamic inversion (Fullea et al. 2021; Xu et al. 2023; Lebedev et al. 2024a) to invert the phase-velocity curves of Rayleigh and Love surface waves averaged over all cratons globally for a complete, multi-property average physical model of cratons. The model, illustrated in the text and provided in its entirety in the supplement, is intended to be a useful reference for geophysical and geochemical studies of continents.

## Data and methods

**Fig. 1:**
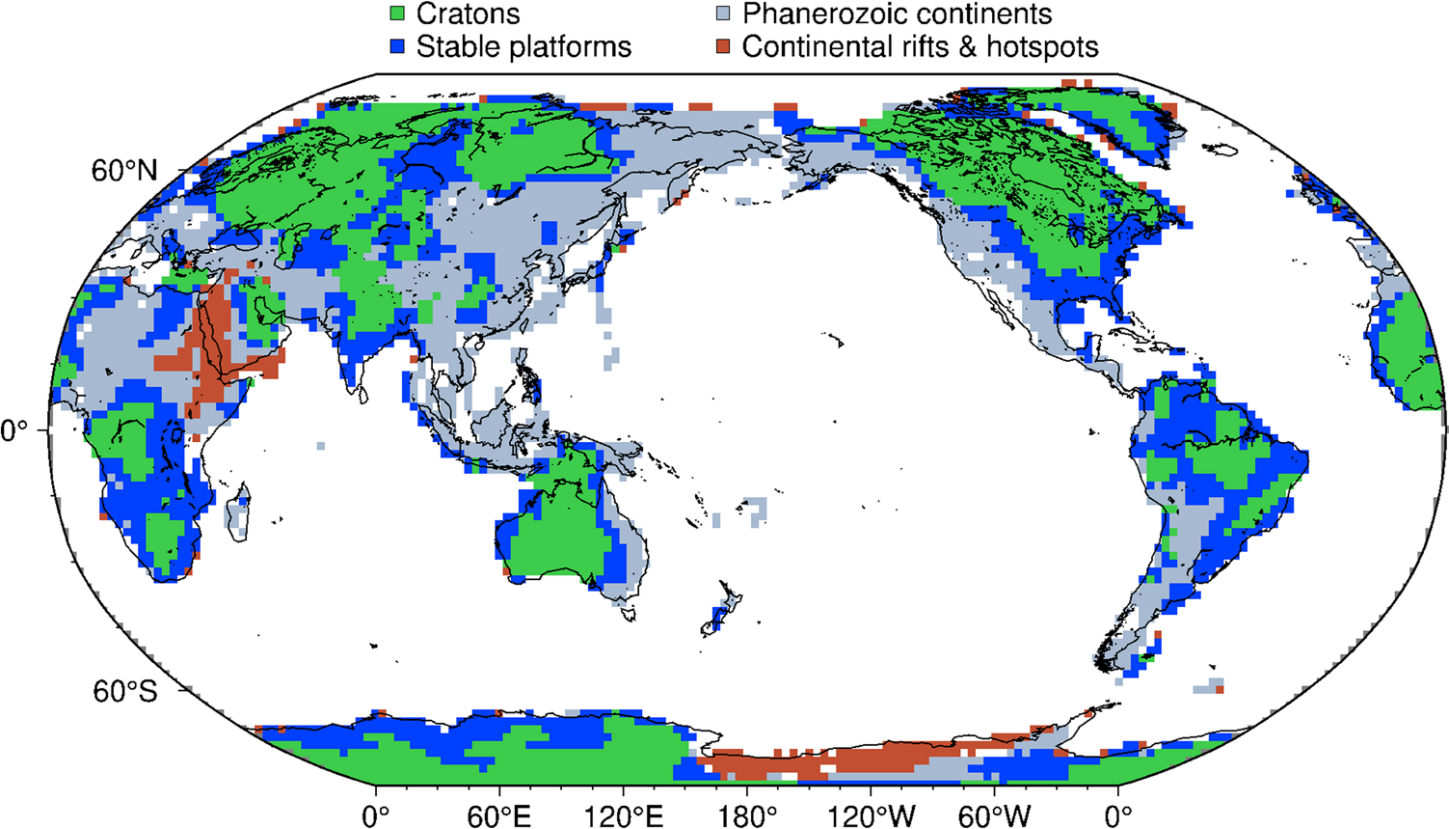
Regionalisation of continents based on the cluster analysis of the global seismic tomography model SL2013 sv (Schaeffer and Lebedev 2015; Civiero et al. 2024). Cratons are coloured green

In this paper, “craton” refers to the regions that have cold and thick lithospheric mantle at the present day (Fig. 1). Specifically, we adopted the boundaries of cratonic regions defined by the regionalisation based on the cluster analysis of the upper mantle tomography model SL2013 sv (Schaeffer and Lebedev 2013, 2015). The cratonic tectonic type in this regionalization covers all the major cratons and excludes those that have been modified and lost their cold, thick lithospheric keels, such as the Sino-Korean Craton (Fig. 1).

The craton-average dispersion curves were computed as the average of the phase-velocity curves at all points of a dense global grid that fall within cratons (Civiero et al. 2024). These phase-velocity curves were obtained from a set of phase-velocity maps, computed by Civiero et al. (2024) at densely spaced periods using very large sets of Rayleigh and Love phase velocities. The phase-velocity tomography mapped both the isotropic-average phase velocities of Rayleigh and Love waves and their azimuthal anisotropy (Lebedev and van der Hilst 2008; Civiero et al. 2024). The azimuthally-isotropic-average phase velocities are thus isolated from the effects of azimuthal anisotropy and can be related to the temperature and thickness of the lithosphere. The average Rayleigh and Love phase-velocity curves extend from 20 to 300 s period and have very small random errors, due to the averaging over many thousands of point curves. Thanks to this, they can constrain the average cratonic structure tightly. We also use craton-average surface heat flow and elevation to constrain the upper-crustal heat production, which has a large effect on the shallow geothermal gradient, and crustal density, respectively. The heat flow data is computed by averaging the samples from the global heat flow map from Jaupart et al. (2015), and the elevation is from the General Bathymetric Chart of the Oceans Grid (GEBCO Bathymetric Compilation Group 2022).

The data are inverted using the thermodynamic inversion of Xu et al. (2023), modified from that of Fullea et al. (2021) so as to enhance the convergence and ensure the lowest possible misfits, which results in a high degree of completeness of the extraction of structural information from the data. The improvements included adding roughness regularisation to the radial anisotropy, calculating P-wave radial anisotropy from that of S waves, normalising the model parameters and fine-tuning their weights in the gradient search, and fine-tuning the gradient-search settings. A key inversion parameter is the depth of the lithosphere-asthenosphere boundary (LAB). In the forward problem, a steady-state geotherm is constructed between the surface and the lithosphere-asthenosphere boundary (LAB) by solving the 1-D conductive heat transfer equation (Fullea et al. 2021):

1 $$\nabla \cdot \left(k\left(z,T,P\right)\nabla T\left(z\right)\right)=-H\left(z\right),$$

where k(z, T, P) is thermal conductivity that changes with pressure and temperature (Hofmeister 1999) and H(z) is the radiogenic heat production. The pertinent parameters are specified in Tables 1 and 2. The temperature at the top of the conductive layer (the Earth’s surface) is set to 0, and the temperature at its bottom (LAB) is set to 1290 °C. At temperatures exceeding 1290 °C, convection is assumed to commence, so that this temperature defines the bottom of the mechanical lithosphere. Four additional temperature variables are defined at sub-lithospheric depths in the upper mantle (Table 2), with linear temperature gradients between these depths. The buffer layer just below the lithosphere has a thermal gradient with a value between that of the lithospheric conductive geotherm and that of the mantle adiabat in the convecting asthenosphere. The buffer layer represents the lowermost part of the Earth’s mantle upper thermal boundary layer, in which the transition from conductive to convective regimes takes place and in which the heat transfer occurs by a mix of conduction and convection (e.g., Fullea et al. 2021). The deepest temperature parameter is at 400 km depth and the remaining two—between the bottom of the buffer and 400 km. The temperature gradients between the sub-lithospheric temperature knots are damped to 0.5 °C/km (Katsura et al. 2010). The conductive geotherm in the lithosphere and the geotherm in the sublithospheric mantle given by the inversion form the 1-D thermal model. The model heat flow is computed using this geotherm and the thermal conductivity given in Table 1.

**Table 1:** Fixed and variable parameters in the crust

Layer	Vs (km/s)	Mass density (10^3^ kg/m^3^)	DOB (km)	Vp/Vs	Thermal conductivity [mW/(m⋅K)]	Radiogenic heat production (μW/m^3^)
Sediment	0.176	2.147	0.176	2.23	2.13	1.66*
Upper crust	3.529	2.731	12.932	1.71	2.13	1.66
Middle crust	3.695	2.810	26.166	1.74	2.33	0.99
Lower crust	3.932	2.928	38.329	1.74	2.10	0.20

^*^The radiogenic heat production values in the Sediment and Upper-crust layers are kept equal

Underscore marks initial values of the variable parameters in the inversion

DOB – depth of the bottom of the layer, relative to the sea level

**Table 2:** Parameters used in the upper mantle

Layer	Parameter	Value	Remarks
Lithospheric mantle	Radiogenic heat production (μW/m^3^)	0.01	
	Thermal conductivity at room conditions [mW/(m⋅K)]	5.3	*T* = 298 K *P* = 1 atm
	Grüneisen parameter*	1.25	*γ*
	Isotherm bulk modulus*	130	*K*_T_
	Derivative of isotherm bulk modulus with respect to pressure*	4.3	d*K*_T_/d*P*
	Al_2_O_3_ (wt%)	0.99	
	FeO (wt%)	6.4	
Lithosphere-asthenosphere boundary	LAB depth (km)	200	
	Temperature at LAB (°C)	1290	
Buffer layer	Temperature at bottom of the layer (°C)	1400	
	Temperature gradient ratio with respect to lithospheric mantle	0.33–0.5	Change linearly with LAB depth
Sublithospheric mantle	Temperature node 1 (°C)	1420	
	Temperature node 2 (°C)	1470	
	Temperature node 3 (°C)	1520	At 400 km depth
	Reference temperature gradient (°C/km)	0.5	
	Al_2_O_3_ (wt%)	4.4	
	FeO (wt%)	8.05	

^*^ These parameters are used to define a P–T-dependent thermal conductivity in the lithospheric mantle (see Eq. 2 in Afonso et al. 2008 for details)

Underscore marks initial values of the variable parameters in the inversion

Temperature nodes 1 and 2 are evenly distributed at depths between the bottom of the buffer layer and 400 km

One-dimensional (1-D) seismic-velocity profiles are then calculated. In the crust, the model is parameterised using four layers with constant parameters within each, the parameters including shear-wave velocity (Vs), density, and the depth of the bottom of the layers (Table 1). The parameters that are not constrained by the data, e.g., the thickness of the sedimentary, the upper and middle crustal layers and the density in the sedimentary and the upper crustal layers, are set to the craton-average values from crust1.0 (Laske et al. 2013; Table 1). Compressional-wave velocity in the crust is coupled to shear-wave velocity (Table 1). In the upper mantle, both seismic velocities and density are calculated from temperature, pressure and composition using computational petrology. The chemical composition is based on the major oxide system CFMAS (CaO-FeO-MgO-Al_2_O_3_-SiO_2_) and the average composition of cratons from Afonso et al. (2008) is used. The composition is fixed during the inversion because the data we use is weakly sensitive to it. The stable mineral assemblage is determined by minimising the Gibbs free energy (Connolly 2005), using the thermodynamic data base of Stixrude and Lithgow-Bertelloni (2011). Seismic velocities and density are calculated from the elastic moduli and density of the constituent stable end-member minerals (Connolly and Kerrick 2002; Afonso et al. 2008). Seismic velocities are then corrected to account for anelasticity (Fullea et al. 2012, 2021, and references therein). In particular, shear-wave velocity V_S_ is corrected by

2 $${V}_{S}={V}_{S0}\left(1-\frac{1}{2}\text{cot}\frac{\pi \alpha }{2}\right){Q}_{S}^{-1}, {Q}_{S}=A{\left[\frac{{T}_{0}}{d}\text{exp}\left(\frac{-\left(E+P{V}^{*}\right)}{RT}\right)\right]}^{\alpha },$$

where T and P are temperature and pressure at given depths, V_S0_ is the unrelaxed high frequency anharmonic velocity computed at T and P with given composition, R is the universal gas constant, the activation energy E = 424 kJ/mol, the activation volume V* = 16 × 10^−6^m^3^/mol, the reference period of seismic waves T_0_ = 50 s, d = 10^4^ μm, α = 0.26, and A = 750 μm^α^/s^α^. Vp is corrected with a similar formula (Fullea et al. 2012).

In the mantle transition zone and shallow lower mantle, shear-wave velocity is parameterised explicitly and damped to that in ak135 (Kennett et al. 1995). The density profile from PREM is adopted below 400 km depth, down to the centre of the Earth. The attenuation in the lower mantle is taken from ak135. Seismic velocities converge to ak135 at ~ 2000 km depth.

Radial anisotropy of S waves (the difference in the velocities of horizontally and vertically polarised shear waves) is explicitly parameterised from the surface to the mantle transition zone and the upper part of the lower mantle, with the deepest basis function decreasing linearly from 1 to 0 between 660 and ~ 2000 km depth. The radial anisotropy is regularised to reduce small-scale oscillations not required by the data. Anisotropy of P waves is calculated from that of S waves, assuming that it is entirely due to that of the shear modulus, with the compressional modulus isotropic. The phase velocities of Rayleigh and Love waves are computed from the profiles of seismic velocity, density, and attenuation using the modes code MINEOS (Masters et al. 2007), modified for the travelling-wave decomposition (Nolet 2008) and for phase-velocity computation speed (Lebedev et al. 2013; Ravenna and Lebedev 2018). The isostatic elevation is computed from the density profile.

To summarise, in addition to the LAB-depth parameter, the unknowns in the inversion include the radiogenic heat production in the upper crust, the isotropic shear wave velocity in the crust, the depth of the crust-mantle boundary (Table 1), temperature in the sublithospheric upper mantle (Table 2), the isotropic shear wave velocity in the mantle transition zone and upper part of the lower mantle, and the radial anisotropy from the surface to the shallow lower mantle. The non-linear inverse problem is solved using the Levenberg–Marquardt method (More et al. 1980). Regularisation is applied to penalise oscillatory vertical variations of the sublithospheric temperature and radial anisotropy. The resulting model comprises the profiles of temperature, pressure, seismic velocities, density, and radial anisotropy in the crust and upper mantle.

## Results

The craton-average model yielded by the thermodynamic inversion matches the observations remarkably well. The difference between the predicted and observed phase velocity dispersion curves (phase velocity as a function of period) is 0.010% (Rayleigh) and 0.019% (Love) on average and peaks at 0.044% (Rayleigh) and 0.055% (Love), at least an order of magnitude smaller than typically obtained in upper mantle surface-wave studies. The small misfits are possible thanks to the very small random errors in the data, due to the averaging over all cratons, and thanks to the fine tuning of the inversion and its convergence (Xu et al. 2023). The close fit to the data is important because this enables full extraction of the subtle signal of the lithospheric structure from the data (Lebedev et al. 2024a). Along with the surface-wave data, the model also fits closely the craton-average heat flow and elevation.

**Fig. 2:**
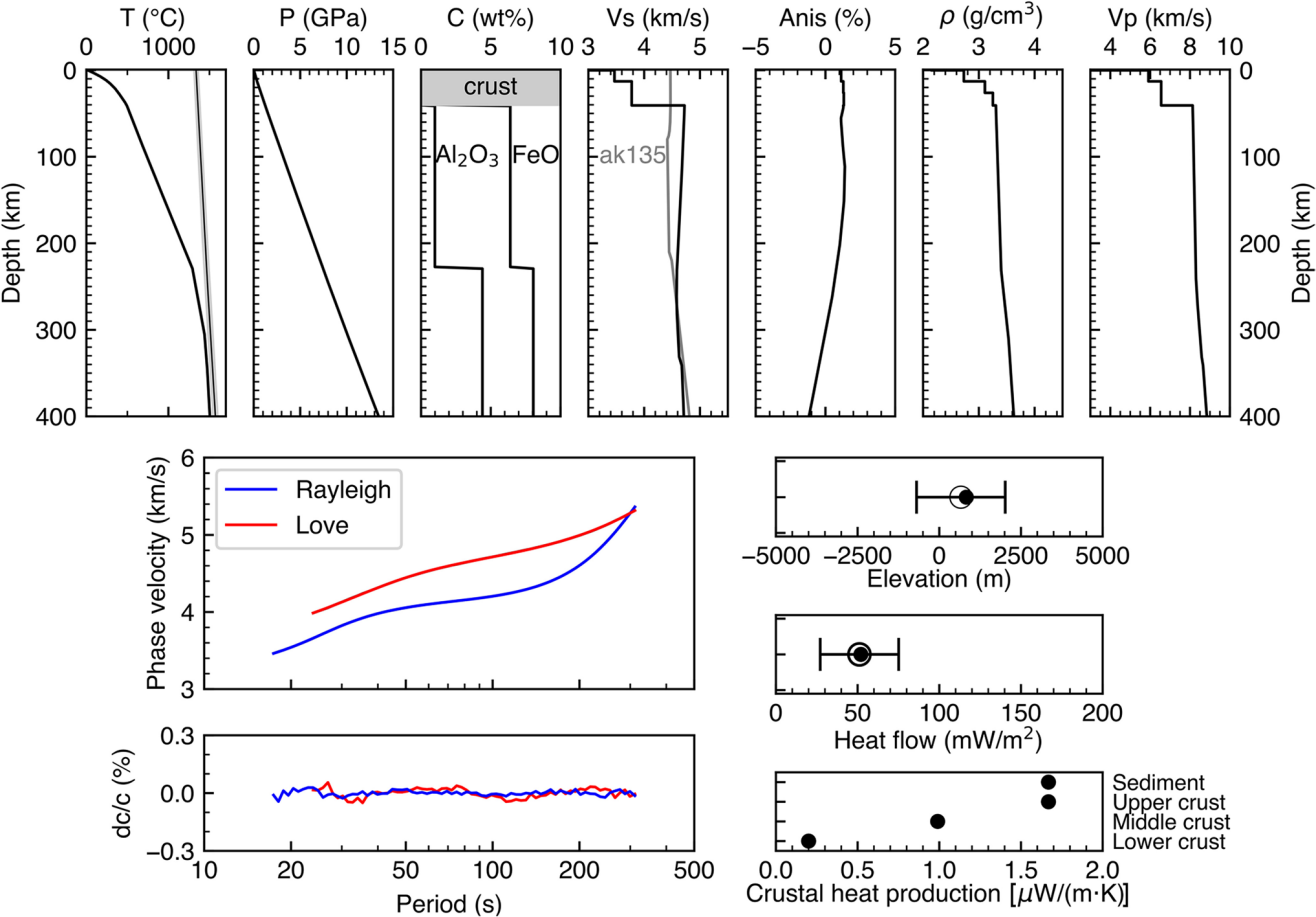
The craton-average model and its fit to the data. (Top) From left to right: depth profiles of temperature, pressure, composition, shear wave velocity, radial anisotropy of shear wave velocity, density, and compressional wave velocity. The composition was assumed, based on average values from the literature, and the other profiles are the result of the inversion. (Bottom left) Phase-velocity curves of Rayleigh and Love waves and the period-dependent misfits, defined as the relative differences between the model-predicted and observed phase velocities. (Bottom right) Predicted and observed elevation (dot and open circle, respectively) and the standard deviation of the observed elevation (error bar); same for heat flow. Radiogenic heat production in the sedimentary layer and the upper, middle, and lower crystalline crust are shown by dots

The cratonic geotherm (temperature as a function of depth) shows a monotonic increase from 0 °C at the surface to 1505 °C (1778.15 K) at the 400 km depth, with the temperature gradient (defined as the derivative of temperature with respect to depth) being the steepest within the crust, due to its high radiogenic heat production and relatively low thermal conductivity (Fig. 2). The gradient gradually decreases around the depth of the Moho and then, again, below the LAB (Fig. 2), where heat transfer by conduction (in the lithosphere) gives way to that by convection (in the underlying mantle).

The craton-average LAB depth is 227.6 km, with the temperature at the LAB postulated to be 1290 °C (1563.15 K). The transitional buffer layer between 227.6 km and 302.8 km depths has a smaller temperature gradient (1.96 °C/km) than in the lithospheric mantle. Below 302.8 km depth, the geotherm is roughly parallel to but slightly colder than the mantle adiabat estimated from laboratory experiments (Katsura et al. 2010), with the average temperature gradient being 0.70 °C/km, which is supported by joint inversion of receiver functions and surface wave datasets (Munch et al. 2025). The pressure increases with depth with an average rate of 0.0334 GPa/km.

**Fig. 3:**
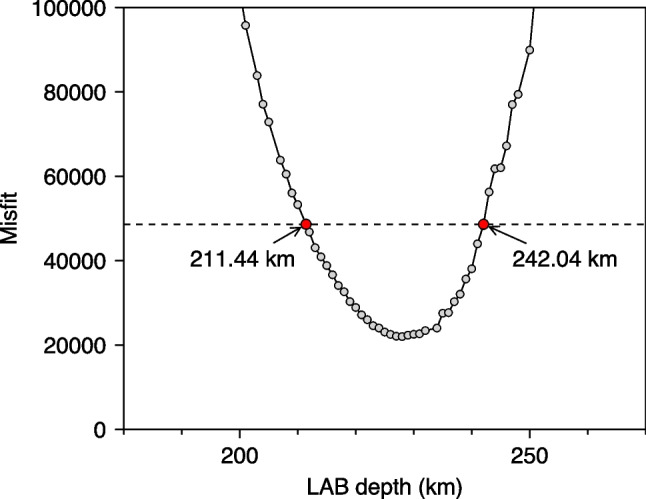
Model-space-projection mapping of the LAB depth uncertainty. Each circle corresponds to one model-space-projection inversion, with the LAB depth fixed at a particular depth. The circle indicates the fixed LAB depth and the misfit of the corresponding inversion. The misfit is computed as ½||***f***||^2^, where ***f*** is a vector consisting of data misfits and the regularisation terms, ||·|| the 2-norm. The horizontal dashed line marks the threshold for the acceptable misfit (see text). The two red dots denote the intersections between the misfit curve and the threshold, which gives the uncertainty range of the LAB depth

The uncertainty of the geotherm in the upper mantle is controlled mainly by the uncertainty of the LAB depth, which we estimated using the model space projection (Bartzsch et al. 2011; Lebedev et al. 2013; Civiero et al. 2024). We repeated the thermodynamic inversion multiple times, each time with the LAB depth fixed at a particular depth (Fig. 3). When the LAB is away from the best-fitting depth, the data misfits increase, even though the trade-offs between all the parameters are allowed to compensate, as much as possible, for the LAB deviation. The threshold for acceptable models is where the misfit reaches the level of systematic data errors added to the misfit of the best-fitting model (Lebedev et al. 2024a). Similar to Civiero et al. (2024), we define the threshold as ½||***f***(***m***_0_)||^2^ + ½||***f***(***m***_0_) – ***f***(***m***_min_)||^2^, where ***f***(***m***_0_) is the vector of data misfits and regularisation terms corresponding to our preferred, craton-average model ***m***_0_, and ***f***(***m***_min_) is the vector corresponding to a very weakly regularised inversion which over-fits the data and noise in the data with an exotic, oscillatory model but makes sure that the remaining misfit represents random phase-velocity errors that cannot be fitted by any dispersion curves. The first term, ½||***f***(***m***_0_)||^2^, represents the misfit of the craton-average model. The second term, ½||***f***(***m***_0_) – ***f***(***m***_min_)||^2^, which is the difference between the best-fitting model and the weakly regularised model, represents an estimate of the level of systematic errors. The procedure yields an LAB-depth uncertainty range of [211.44, 242.04 km]. The envelope of the bundles of geotherms within this range provides an estimate for the uncertainty of the geotherm within the upper mantle, which gives uncertainties of ± 45 °C, ± 65 °C, ± 50 °C at 100 km, 200 km, and 300 km depth, respectively.

**Fig. 4:**
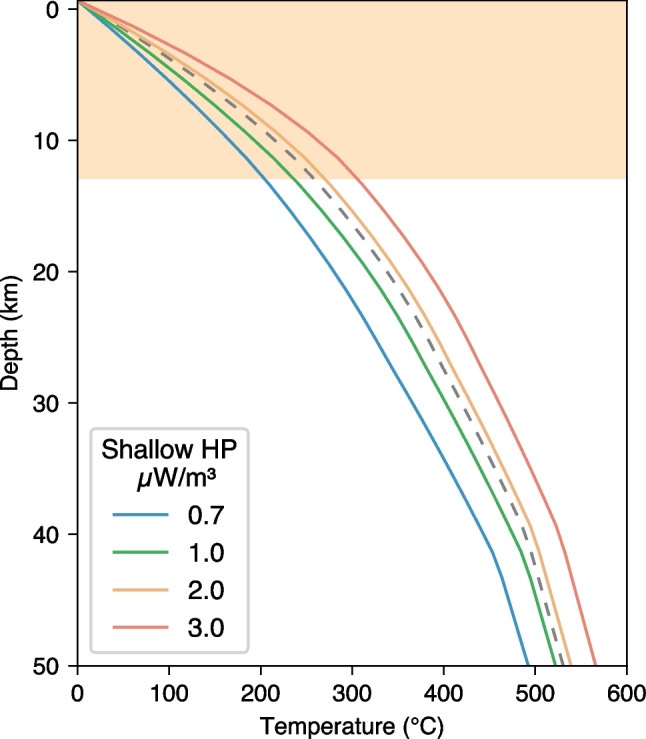
Crustal geotherms yielded by thermodynamic inversions with different radiogenic heat production in the shallow crustal layers, i.e. the sediment and the upper crystalline crust. The dashed line is the craton-average geotherm. The orange shaded area indicates the depth range of the sedimentary layer and upper crust. The heat production in the rest of the crust and mantle are the same in all the inversions

Another substantial component of the uncertainty of the geotherm is due to the unknown distribution of the radiogenic heat production in the crust (e.g., Jaupart et al. 2015; Chambers et al. 2023). We estimate this effect by varying the heat production in the sediments and upper crust in the broad range between 0.7 and 3.0 μW/m^3^ (Fig. 4). We use the region bounded by the geotherms with heat production of 0.7 and 3.0 μW/m^3^ as a conservative quantitative estimation of the uncertainty.

**Fig. 5:**
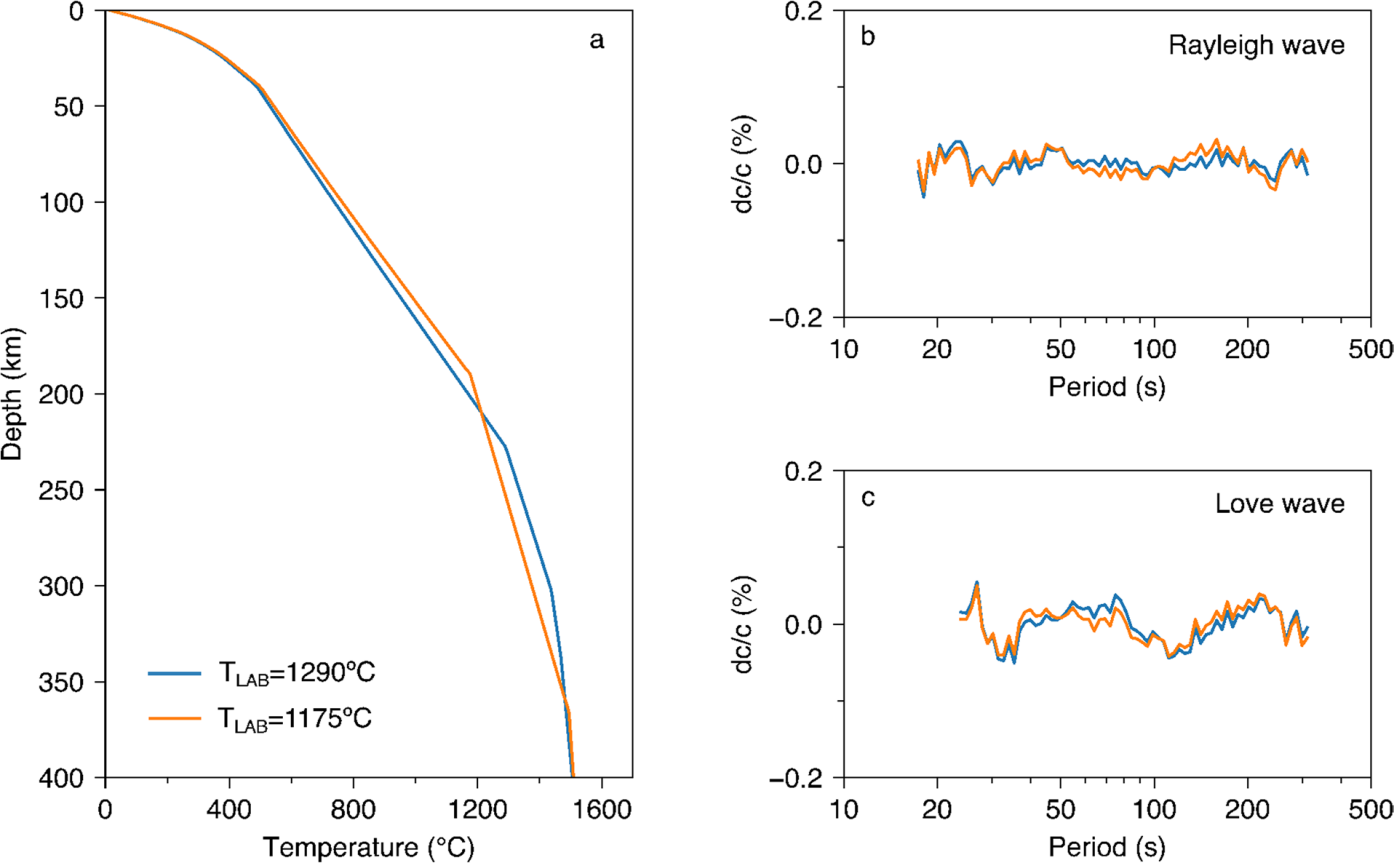
The effect of T_LAB_ (assumed temperature that defines the bottom of the lithosphere) on the craton-average geotherms and the data fit. (**a**) Geotherms inverted with T_LAB_ = 1175 and 1290 °C, respectively, and the associated data misfits for (**b**) Rayleigh and (**c**) Love phase velocity data

Uncertainties in other model parameters can also affect the best-fitting LAB depth. We assumed the temperature at the LAB (T_LAB_) to be 1290 °C, but some authors assume it to be lower, as low as 1175 °C (Hoggard et al. 2020). We repeat our thermodynamic inversion with the lower postulated LAB temperature of T_LAB_ = 1175 °C and find that the resulting model fits the data as well as that with T_LAB_ = 1290 °C (Fig. 5). The colder LAB has to be shallower, the lithospheric temperature increases slightly, and the buffer layer becomes thicker and colder, so as to compensate for the warmer lithosphere. The T_LAB_ values of 1175 °C and 1290 °C are close to the lowest and highest temperature endmembers found in the literature (e.g., Hoggard et al. 2020; Fullea et al. 2021), and we use the corresponding models to estimate the full range of the uncertainty of the average cratonic geotherm.

**Fig. 6:**
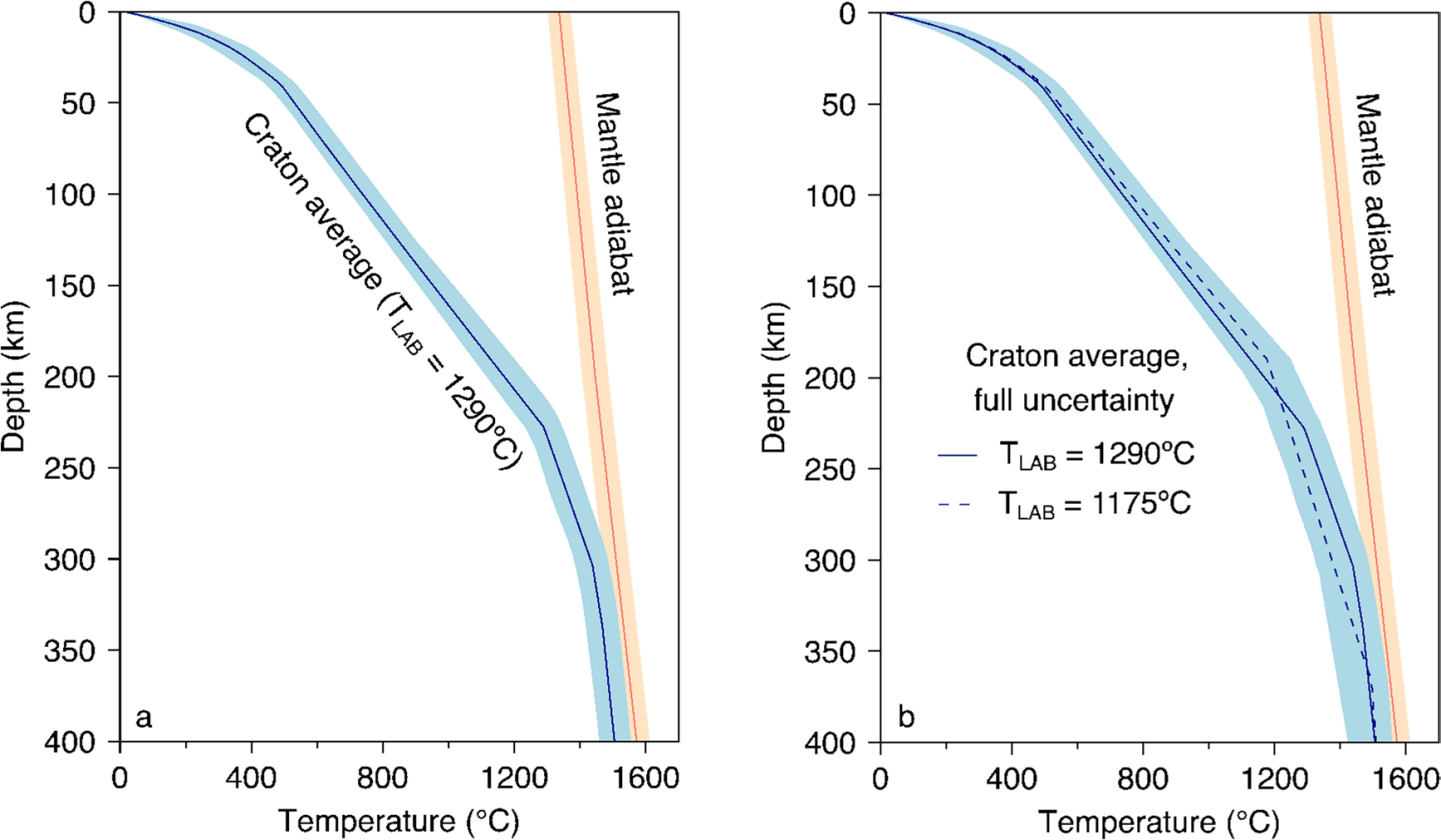
Craton-average geotherm (blue) and its uncertainty (blue-shaded area). (**a**) Uncertainty assuming T_LAB_ = 1290 °C; (**b**) Uncertainty assuming T_LAB_ can vary between 1175 °C and 1290 °C, which covers the range of T_LAB_ used in the literature. The orange line and shaded area denote the mantle adiabat of Katsura et al. (2010) and its uncertainty, respectively

The LAB depth, heat-production uncertainties, and T_LAB_ uncertainties are combined to produce the complete uncertainty of the craton-average geotherm (Fig. 6). The assumed composition of the mantle lithosphere also affects the computed geotherms. However, the effect of plausible changes in the degree of depletion of the lithospheric rock is small (e.g., Fullea et al. 2021), well within our uncertainty estimate (Fig. 6).

The seismic-velocity profiles are constrained by our data down to the bottom of the mantle transition zone at 660 km depth (see also Civiero et al. 2024, for the sensitivity analysis of phase velocities in a period range as we use here). In the lower mantle below, they converge gradually to ak135 (see supplementary materials).

Within the upper mantle, shear wave speed monotonically decreases with depth from the Moho to the LAB, stays approximately constant in the buffer layer and increases with depth below. The craton-average Vs is significantly higher than the global average (e.g., Dziewonski and Anderson 1981; Kennett et al. 1995), as has long been observed in seismic studies.

Radial anisotropy shows that V_SH_, the speed of horizontally polarized shear waves, is ~ 1% faster than the V_SV_, vertically polarized shear waves, above 150 km depth, in cratons on average. The anisotropy then decreases to 0% (V_SH_ = V_SV_) around 300 km, flips sign and reaches −1% (V_SH_ < V_SV_) at 400 km depth.

## Discussion

**Fig. 7:**
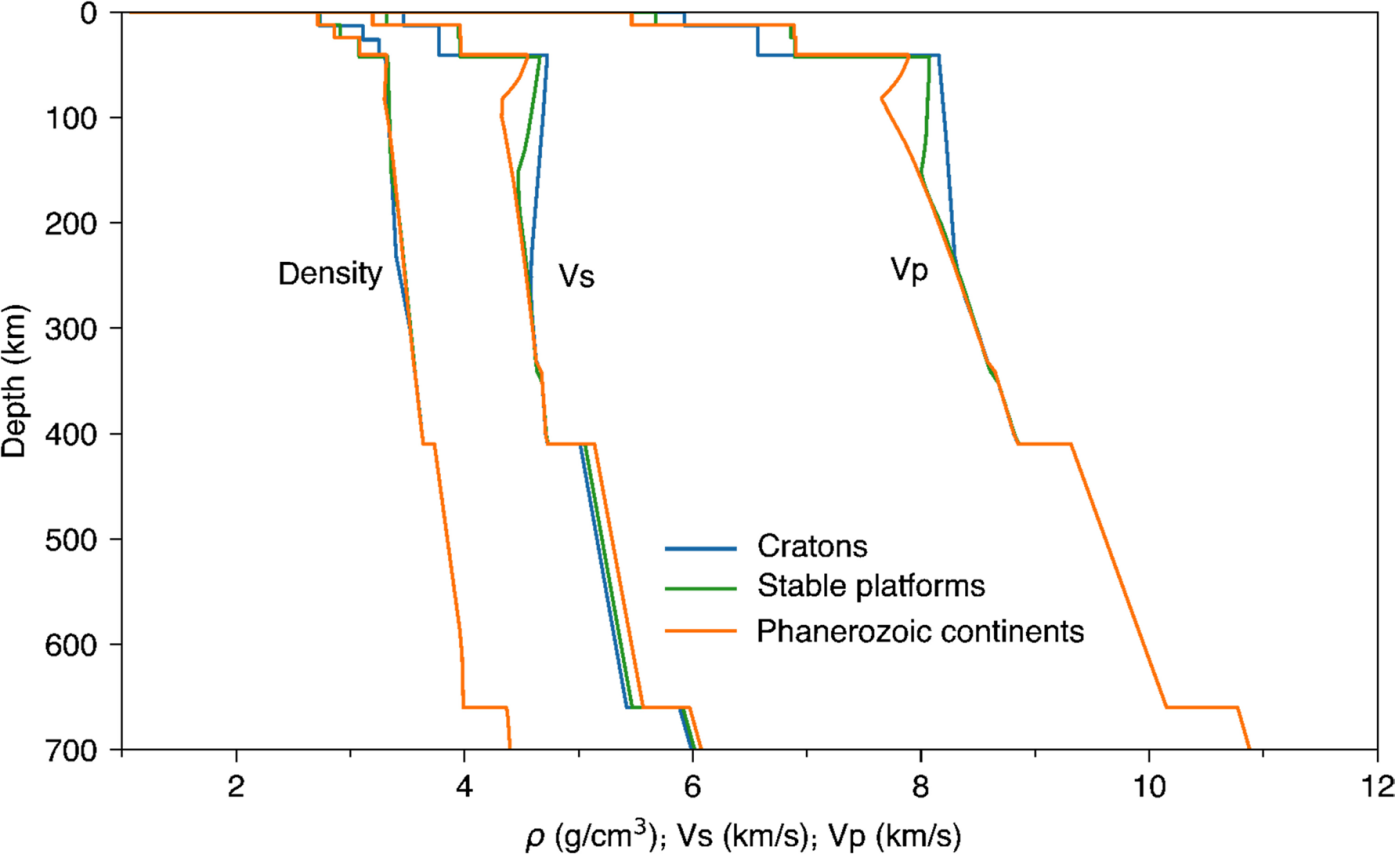
Comparison of the density and seismic velocity (Vs and Vp) profiles that are averages over different continental tectonic environments: cratons, stable platforms, and Phanerozoic continents. The definition of the tectonic environments follows the tomography-based tectonic regionalisation in Fig. 1

The density of the depleted lithospheric mantle beneath cratons has been proposed to be similar to that elsewhere at the same depths, because the depletion-induced density reduction is compensated by the density increase caused by the cold geotherm—this is known as the isopycnicity hypothesis (e.g., Jordan 1978). The hypothesis has been questioned recently in regional-scale and global studies (e.g., Artemieva et al. 2019; Wang et al. 2022). We compare the craton-average density profile with the average density profiles of two other type continental environments (Fig. 7). The models for the other tectonic environments were computed using the thermodynamic inversion of the average phase-velocity curves for those environments (Xu et al. 2023; Civiero et al. 2024), defined using the tomography-based tectonic regionalisation (Fig. 1). The three continental regions have very similar density profiles. By contrast, seismic velocities (Vp and Vs), which are strongly sensitive to temperature and less sensitive to composition, show large differences due to the temperature variations. Our model thus confirms that the stable continental regions are approximately isopycnic, on average, which, however, does not preclude local deviations from the average.

Mantle xenoliths provide valuable information on the temperature distribution in the upper mantle that is independent and complementary to that from geophysical data (e.g., Boyd et al. 1985; Kopylova et al. 1999; Pearson et al. 2021). Here, we compare our model to two compilations of xenolith measurements, taken from (1) McKenzie et al. (2005) and Priestley and McKenzie (2006) and (2) Garber et al. (2018).

**Fig. 8:**
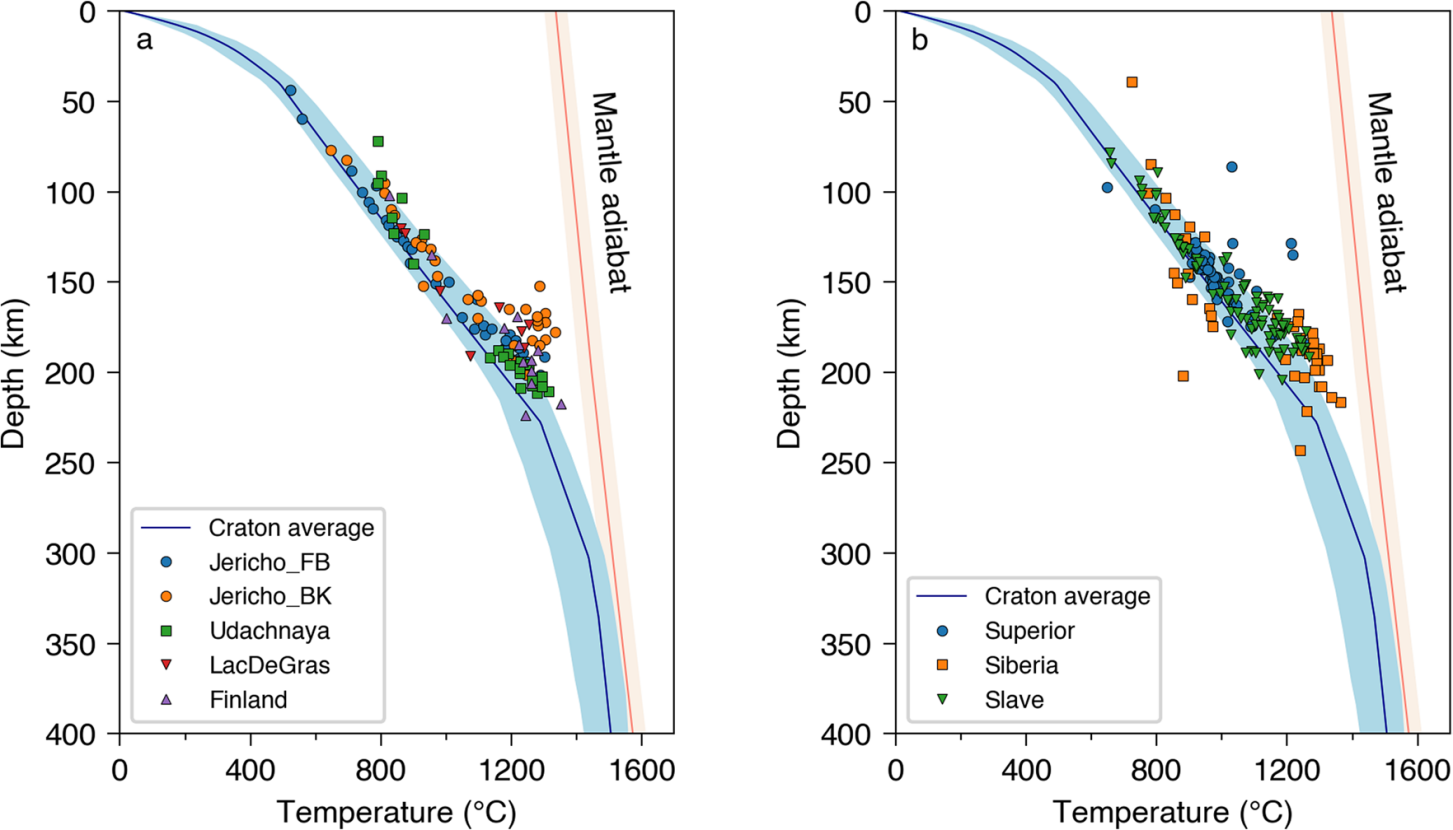
Comparison of the craton-average geotherm (blue solid line) and depth-temperature estimates from xenoliths taken from the compilations of (**a**) McKenzie et al. (2005) and Priestley and McKenzie (2006) and (**b**) Garber et al. (2018). The blue-shaded area indicates the uncertainty of the geotherm, as estimated in Fig. 6b. Jericho_FB and Jericho_BK are two sets of estimates from the Jericho kimberlite in northern Canada (McKenzie et al. 2005 and references therein). The mantle adiabat (orange line) and its uncertainty (orange-shaded area) are from Katsura et al. (2010)

The depth-temperature distributions obtained from both datasets show a strong match with the geotherm in our model, computed completely independently (Fig. 8). Nearly all the xenolith data plot within the depth range of the lithospheric mantle in our craton-average model.

Interesting patterns of differences are also observed between the geophysically constrained geotherm and the xenoliths. Rather than running through the middle of the cluster of xenoliths, the craton-average geotherm tends to delineate the lower boundary of the cloud of points of the xenolith measurements. A notable exception is presented by the scatter of xenolith measurements from Siberia, which may be due to errors in the data or, alternatively, indicate large spatio-temporal variations in the lithospheric mantle temperature.

Above 150 km depth, the majority of xenoliths measurements are very close to the craton-average geotherm, within its uncertainty. Below 150 km depth, the pattern changes: most xenolith measurements indicate temperatures 100–200 °C higher than the craton-average geotherm.

One explanation for this systematic pattern is that the xenolith data tend to overestimate the temperature in the lower part of the lithosphere. P–T estimates from xenoliths can be less robust when T > 1100 °C and P > 5 GPa (Smith 1999), which corresponds the depth > 150 km. Alternatively, xenoliths may produce higher temperature estimates when sampling a non-equilibrium, transient state of the mantle (Pearson et al. 2014). The systematic difference poses important questions that warrant further investigation in future studies.

## Conclusions

Thermodynamic inversion of average surface-wave data from all of the Earth’s cratons yields a self-consistent 1-D physical model of average cratonic lithosphere. The model matches, with exceptionally small misfits, the craton-average phase velocities of surface waves, as well as surface heat flow and elevation. It comprises the vertical distributions of temperature (the geotherm), pressure, velocities of shear (Vs) and compressional (Vp) waves, radial anisotropy of shear wave velocity, and density. Within the upper mantle, the distributions of these parameters are calculated from temperature and composition by means of computational petrology in the course of the thermodynamic inversion and are, thus, fully mutually consistent. The conductive geotherm in the lithosphere transitions to a mantle adiabat in the convecting sub-lithospheric mantle, with the mantle adiabat slightly cooler than previously published global-average estimates (Katsura et al. 2010; Katsura 2022). Representative cratonic mantle-lithosphere composition is assumed and is fully consistent with the data, with no compositional layering in the mantle lithosphere required to explain the geophysical data (Davison et al. 2023).

Comparisons of cratonic and non-cratonic density profiles confirms the isopycnic hypothesis of Jordan (1978). Based on the published average lithospheric compositions and the geotherms constrained by our thermodynamic inversion of seismic data, the average density profile of the sub-cratonic upper mantle is very similar to those beneath stable platforms and Phanerozoic tectonic continents.

Independent mantle-xenolith measurements show a close match with the geophysically derived craton-average geotherm down to 150 km depth. Below 150 km, many xenolith measurements show temperatures 100–200 °C higher than the craton-average geotherm, which poses intriguing questions for multi-disciplinary future studies on lithospheric evolution and kimberlite dynamics.

## Supplementary Information

Below is the link to the electronic supplementary material.

Supplementary file1 (TXT 16 KB)

Supplementary file2 (TXT 12 KB)
